# Early clearance of serum HE4 and CA125 in predicting platinum sensitivity and prognosis in epithelial ovarian cancer

**DOI:** 10.1186/s13048-020-00759-9

**Published:** 2021-01-04

**Authors:** Yan Rong, Li Li

**Affiliations:** 1 Department of Gynecologic Oncology, Guangxi Medical University Cancer Hospital, 71 Hedi Road, Nanning, Guangxi 530021 People’s Republic of China; 2 Key Laboratory of Early Prevention and Treatment of Regional High-Incidence Tumors, Ministry of Education, No.22 Shuangyong Road, Nanning, 530021 People’s Republic of China

**Keywords:** Platinum sensitivity, Prognosis, HE4, CA125, Clearance, Chemotherapy

## Abstract

**Objectives:**

To assess the clinical value of early clearance of HE4 and CA125 for platinum sensitivity and prognosis in patients with ovarian cancer.

**Method:**

HE4 and CA125 value including clinical data of 89 patients with ovarian cancer were collected. The clearance of HE4 and CA125 were assessed base on the platinum sensitivity, two-year PFS, PFS and OS.

**Results:**

Sixteen patients were classified as platinum resistant and 73 as platinum sensitive according to the response to platinum-base chemotherapy. When HE4 clearance after 3rd cycle chemotherapy or CA125 clearance after 1st cycle chemotherapy, it gave the highest AUC of 0.788, with 100% of sensitivity and 57.5% of specificity respectively between platinum resistant and platinum sensitive group. In addition, 59 patients were classified as two-year PFS group and 30 as not achieved two-year PFS group according to obtaining two-year PFS or not. It gave the highest AUC of 0.730, with 83.3% of sensitivity and 62.7% of specificity respectively when HE4 clearance after 3rd cycle chemotherapy or CA125 clearance after 1st cycle. The prolonged PFS and OS were significantly associated by the clearance of HE4 after 3rd cycle chemotherapy (*p*< 0.0001, *p*< 0.0001) as well as CA125 after 1st cycle chemotherapy (*p*< 0.0001, *p*< 0.0001).

**Conclusions:**

Our data suggested that the early clearance of HE4 and CA125 could predict platinum response and prognosis in patients with ovarian cancer. Monitoring the HE4 and CA125 during first-line chemotherapy might be helpful in predicting platinum sensitivity and risk to progress and relapse.

## Introduction

Epithelial ovarian cancer (EOC) is the leading cause of mortality among gynecological cancer [2]. Due to the lack of typical clinical performance and reliable screening methods, approximately 75% of patients were diagnosed at advance disease with a poor prognosis. 30–50% of advanced patients will relapse within 5 years after standard surgery and platinum-based chemotherapy. Although the inhibition of the poly ADP-ribose polymerase has become an attractive therapeutic strategy in patients of epithelial ovarian cancer, early identification of drug-resistant or high-risk patients and taking hierarchical management is still crucial for improving prognosis.

Since patients with EOC have no measurable target lesion after initial surgery, it is difficult to assess the treatment response with gynecological examination and image. Effort has been made to find out reliable biomarker for monitoring therapeutic response and detecting relapse in EOC. Cancer antigen 125 (CA125) is the most common serum biomarker for judging treatment response and monitoring recurrent in EOC. Our previous study demonstrated that the median PFS and OS of patients with serum CA125 who had a logarithmic decrease or a decrease to normal within 1 month after treatment were better than those of with a non-logarithmic decrease or a decrease to normal that took longer than 1 month [25]. However, the single role of serum CA125 in predicting prognosis and platinum sensitivity still remains controversial [7, 17, 22]. Therefore, there is still an urgent need to find more promising biomarkers for monitoring the prognosis of ovarian cancer.

Human epididymis protein 4 (HE4), has been proved to be a reliable biomarker for detecting ovarian cancer with a sensitivity of 76% (95%CI, 0.72–0.80) and a specificity of 94% (95%CI, 0.90–0.96) [29] and approved by the Food and Drug Administration in Unite State as a novel tumor biomarker for the diagnosis of ovarian cancer. The exploration of the value of this glycoprotein in predicting prognosis in ovarian cancer is still ongoing [6, 10, 15, 16]. But there are seldom study concern with the correlation of early clearance of serum HE4 combined with CA125 during first-line treatment with platinum sensitivity and prognosis. The aim of this study is to evaluate the role of early clearance of HE4 and CA125 in predicting platinum sensitivity and prognosis in epithelial ovarian cancer.

## Materials and methods

### Patients and clinical data

The retrospective study was conducted in the Guangxi Medical University Cancer Hospital of china from July 2012 to December 2018. Patients diagnosed with epithelial ovarian cancer by histopathology with full serum HE4 and CA125 value and clinical record were available for review. Inclusion criteria were: 1) Initially treated with staging surgery or optimal cytoreductive surgery, including total hysterectomy, bilateral oophorectomy and salpingectomy, peritoneal washing, omentectomy, pelvic/para-aortic nodal dissection, and multiple peritoneal biopsies (multivisceral resection including en bloc resections with bowel resection, upper abdominal procedures, and extensive peritonectomy are required to achieve optimal tumor debulking when necessary). 2) Patients with stage IC and higher stage received platinum-based combined chemotherapy for 6–8 cycles after surgery. 3) Good nutritional status without other cancer. Exclusion criteria were:1) Death due to non-oncological reasons. 2) Lacking of follow-up after treatment.

Responds to treatment and progression were evaluated according to the guidelines of the Gynecology Cancer Intergroup (GCIG) [23] and the Response Evaluation Criteria in Solid Tumors (RECIST) criteria [11]. PFS was defined as the length of time between the initial treatment to the occurrence of the progress or relapse. OS was defined as the length of time between the initial treatment to death. According to the respond to chemotherapy, patients with recurrence within 6 months after the completion of first-line platinum chemotherapy were defined as platinum resistant. Patients who developed recurrence with an interval > 6 months were defined as platinum sensitive patients [8].

The study was compliant with the Declaration of Helsinki, and approved by the Ethics Committee of Guangxi Medical University Cancer Hospital. Informed consents of all treatments and examinations have been obtained from patients or their families.

### Test of HE4 and CA125 value

Serum HE4 and CA125 concentration of each patient were measured at the time of pretreatment, each post-chemotherapy and recurrence. The tests were performed using an electro-chemiluminescence immunoassay (Roche, Diagnostics, Inc., Mannheim, Germany, performed according to the manufacturer’s specifications) at the departmental laboratory of Guangxi Medical University Cancer Hospital. The normal value range of HE4 is less than or equal to 70 pmol/L as suggested by Moore et al. [19], and the normal value of CA125 is less than or equal to 35 U/ml.

### Statistical analyses

All the statistical analyses were conducted by SPSS 25.0 Software. T-test was used for the comparison between groups. The area under the curve (AUC) was used to calculate with a receiver operating characteristics (ROC) in predicting platinum sensitivity and two-year survival. The Kaplan-Meier survival curve and long rank test were used to assess the influence of HE4 and CA125 on PFS and OS. Cox regression models was used to conduct the univariate and multivariate analyses. A two-tailed probability of *p* < 0.05 was defined as a statistically significant.

## Results

### Patients’ characteristics

A total of 89 patients with EOC were included in the study. All patients were followed up to December 31, 2019. At the end of follow-up period, 36 patients were progress, 19 patients were dead and 70 patients were still alive. The median follow-up time was 35 months. Patients’ clinicopathological characteristics and the mean pretreatment level of HE4 and CA125 were presented in Table 1. The mean HE4 value of pretreatment was significantly increased with FIGO stage (*p*=0.008) and tumor grade (*p*=0.000). While the mean CA125 level of pretreatment was only significant increased with FIGO stage (*p*=0.008) and histology types (*p*=0.040). However, there were no significant differences in menopausal, platinum response and two-year PFS with pretreatment HE4 or CA125.

**Table 1:** Characteristics of patients with ovarian cancer and comparison with pretreatment HE4 and CA125 level

Characteristics	Pretreatment HE4 Mean (range) [pmol/L]	Pretreatment CA125 Mean (range) [U/ml]
Menopausal		
Premenopausal, *n*=35	408.8 (32.4–1529)	939.3 (9.0–5023)
Postmenopausal, *n*=54	548.7 (47.4–4703)	855.4 (8.0–4423)
*P* value	0.309	0.733
FIGO stage		
I and II, *n*=37	286.7 (32.4–1500)	543.8 (8.0–3066)
III and IV, *n*=52	641.0 (63.11–4703)	1133.5 (18.8–5023)
*P* value	0.008	0.008
Tumor grade		
1, *n*=11	141.2 (33.6–437)	472.0 (9.0–2062)
2 and 3, *n*=78	543.4 (32.4–4703)	947.1 (683.6–1210)
*P* value	0.000	0.191
Histology		
Serous, *n*=48	618.5 (47.4–4703)	1152.6 (8.0–5023)
Mucinous, *n*=7	232.5 (40.8–1132)	139.7 (12.5–523)
Clear cell, *n*=11	193.9 (32.4–675)	229.4 (13.0–653)
Endometrioid, *n*=8	635.7 (33.6–1529)	714.9 (9.0–1974)
Others, *n*=15	360.2 (81.4–1234)	967.9 (11.68–3643)
*P* value	0.153	0.040
Platinum response		
Sensitive, *n*=73	487.9 (32.4–4703)	955.2 (8.0–5023)
Resistant, *n*=16	520.2 (63.11–1500)	583.5 (15.6–2880)
*P* value	0.854	0.233
Two-year PFS		
YES, *n*=59	389.7 (32.4–2106)	898.6 (7.98–5023)
NO, *n*=30	698.1 (63.11–4703)	868.2 (15.6–3643)
*P* value	0.076	0.905

### The predictive value of HE4 and CA125 in platinum sensitivity

In the analyzed patients, 16 were defined as platinum resistant and 73 as platinum sensitive. The capability of HE4 and CA125 clearance after 1st, 3rd, 6th cycle chemotherapy to predict platinum sensitivity were assessed by ROC and AUC. The clearance was defined as the level of HE4/CA125 reduced to normal value or had a reduction rate of 90% at least. The early clearance was defined as the clearance of HE4/CA125 before the 4th cycle of adjuvant chemotherapy. According to the definition, we found in platinum sensitive group that the clearance of HE4 in 55 of 73 (75.3%) cases after 1st cycle chemotherapy, in 59 of 73 (80.8%) cases after 3rd cycle chemotherapy, and in 61 of 73 (83.6%) cases after 6th cycle chemotherapy. The HE4 non-clearance patients of platinum resistant were found in 10 of 16 (62.5%) cases after 1st cycle chemotherapy, in 12 of 16 (75.0%) cases after 3rd cycle chemotherapy, and in 10 of 16 (62.5%) cases after 6th cycle chemotherapy. The maximum AUC was 0.779 for HE4 after 3rd cycle chemotherapy, with 75.0% of sensitivity, 80.8% of specificity, and a *p* value of 0.000. The maximum AUC was 0.731 for CA125 after 1st cycle chemotherapy, reporting 75.0% of sensitivity, 71.2% of specificity, and a *p* value of 0.004. When the two biomarkers were combined, the result showed that when HE4 clearance after 3rd cycle chemotherapy or CA125 clearance after 1st cycle chemotherapy, it gave the highest AUC of 0.788, with 100% of sensitivity and 57.5% of specificity respectively. When HE4 clearance after 3rd cycle chemotherapy and CA125 clearance after 1st cycle chemotherapy were used at the same time, the AUC, sensitivity, and specificity were 0.723, 50, and 94.5% respectively. The AUC, sensitivity, specificity, positive predictive value (PPV), negative predictive value (NPV), accuracy and *p*-value base on each parameter were shown in Table 2.

**Table 2:** Classification base on the platinum response and the AUC value using the clearance of HE4 and CA125

	Platinum sensitive	Platinum resistant	Sensitivity	Specificity	PPV	NPV	Accuracy	*P*-value	AUC
	HE4 clearance	HE4 non-clearance							
1 cycle	55/73 (75.3%)	10/16 (62.5%)	62.5%	75.3%	35.7%	90.2%	73.0%	0.018	0.689
3 cycle	59/73 (80.8%)	12/16 (75.0%)	75.0%	80.8%	54.5%	93.7%	79.8%	0.000	0.779
6 cycle	61/73 (83.6%)	10/16 (62.5%)	62.5%	83.6%	45.5%	91.0%	79.8%	0.004	0.730
	CA125 clearance	CA125 non-clearance							
1 cycle	52/73 (71.2%)	12/16 (75.0%)	75.0%	71.2%	36.4%	92.9%	71.9%	0.004	0.731
3 cycle	69/73 (94.5%)	6/16 (37.5%)	37.5%	94.5%	60.0%	87.3%	84.3%	0.046	0.660
6 cycle	71/73 (97.3%)	6/16 (43.8%)	43.8%	97.3%	77.8%	88.8%	87.6%	0.011	0.705
	HE4 or CA125 clearance	Both HE4 and CA125 non-clearance							
3 cycle of HE4 or 1 cycle of CA125	42/73 (57.5%)	16/16 (100%)	100.0%	57.5%	34.0%	100.0%	65.2%	0.000	0.788
	Both HE4 and CA125 clearance	HE4 or CA125 non-clearance							
3 cycle of HE4 and 1 cycle of CA125	69/73 (94.5%)	8/16 (50.0%)	50.0%	94.5%	66.7%	89.6%	86.5%	0.005	0.723

### The clearance of serum HE4 and CA125 in predicting two-year PFS

In this part (Table 3), 59 patients were classified as two-year PFS group and 30 as not achieved two-year PFS group according to obtaining two-year PFS or not. Using the predefined standard with the clearance of HE4 and CA125, we found in two-year PFS group that the clearance of HE4 in 45 of 59 (76.3%) cases after 1st cycle chemotherapy, in 50 of 59 (84.7%) cases after third cycle chemotherapy, and in 51 of 59 (86.4%) cases after sixth cycle chemotherapy. The HE4 non-clearance patients of not achieved two-year PFS were found in 14 of 30 (46.7%) cases after 1st cycle chemotherapy, in 17 of 30 (56.7%) cases after 3rd cycle chemotherapy, and in 14 of 30 (46.7%) cases after 6th cycle chemotherapy. We found a statistically significant difference in predicting two-year PFS between patients with HE4 clearance and non-clearance at the period of after the 3rd and 6th cycle chemotherapy (*p*=0.001, 0.011), and the AUC of 0.707, 0.666 respectively (Table 3). The early clearance of HE4 after 3rd cycle chemotherapy demonstrated the optimal accuracy of 75.3%, with a corresponding 56.7% of sensitivity and 84.7% of specificity (PPV=65.4%, NPV=79.4%). The CA125 clearance profile in two-year PFS group were found in 43 of 59 (72.9%) cases after 1st cycle chemotherapy, in 56 of 59 (94.9%) cases after 3rd cycle chemotherapy, and in 57 of 59 (96.6%) cases after 6th cycle chemotherapy. The CA125 non-clearance patients of not achieved two-year PFS group were found in 17 of 30 (56.7%) cases after 1st cycle chemotherapy, in 7 of 30 (23.3%) cases after 3rd cycle chemotherapy, and in 7 of 30 (23.3%) cases after 6th cycle chemotherapy. Significant differences in predicting two-year PFS was only found in patients with CA125 clearance after the 1st cycle chemotherapy (*p*=0.023), with the AUC of 0.648, and a corresponding 56.7% of sensitivity and 72.9% of specificity (PPV=51.5%, NPV=76.8%). When HE4 and CA125 were combined, it was shown that the AUC reached 0.730 when the HE4 value after 3rd cycle chemotherapy or the CA125 after 1st cycle chemotherapy that declined above 90% or normalized (*p*=0.000). However, when both of the HE4 after 3rd cycle chemotherapy and the CA125 after 1st cycle chemotherapy declined above 90% or normalization, the AUC was 0.625 with *p*=0.056.

**Table 3:** Classification base on the two-year PFS and the AUC value using the clearance of HE4 and CA125

	Two-year PFS		Sensitivity	Specificity	PPV	NPV	Accuracy	*P*-value	AUC
	Yes	No							
	HE4 clearance	HE4 non-clearance							
1 cycle	45/59 (76.3%)	14/30 (46.7%)	46.7%	76.3%	50.0%	73.8%	66.3%	0.078	0.615
3 cycle	50/59 (84.7%)	17/30 (56.7%)	56.7%	84.7%	65.4%	79.4%	75.3%	0.001	0.707
6 cycle	51/59 (86.4%)	14/30 (46.7%)	46.7%	86.4%	63.6%	76.1%	73.0%	0.011	0.666
	CA125 clearance	CA125 non-clearance							
1 cycle	43/59 (72.9%)	17/30 (56.7%)	56.7%	72.9%	51.5%	76.8%	67.4%	0.023	0.648
3 cycle	56/59 (94.9%)	7/30 (23.3%)	23.3%	94.9%	70.0%	70.9%	70.8%	0.161	0.591
6 cycle	57/59 (96.6%)	7/30 (23.3%)	23.3%	96.9%	77.8%	71.3%	71.9%	0.126	0.600
	HE4 or CA125 clearance	Both HE4 and CA125 non-clearance							
3 cycle of HE4 or 1 cycle of CA125	37/59 (62.7%)	25/30 (83.3%)	83.3%	62.7%	53.2%	88.1%	69.7%	0.000	0.730
	Both HE4 and CA125 clearance	HE4 or CA125 non-clearance							
3 cycle of HE4 and 1 cycle of CA125	56/59 (94.9)	9/30 (30.0%)	30.0%	94.9%	75.0%	72.7%	73.0%	0.056	0.625

### The relationship between prognosis and the serum HE4 and CA125

**Fig. 1:**
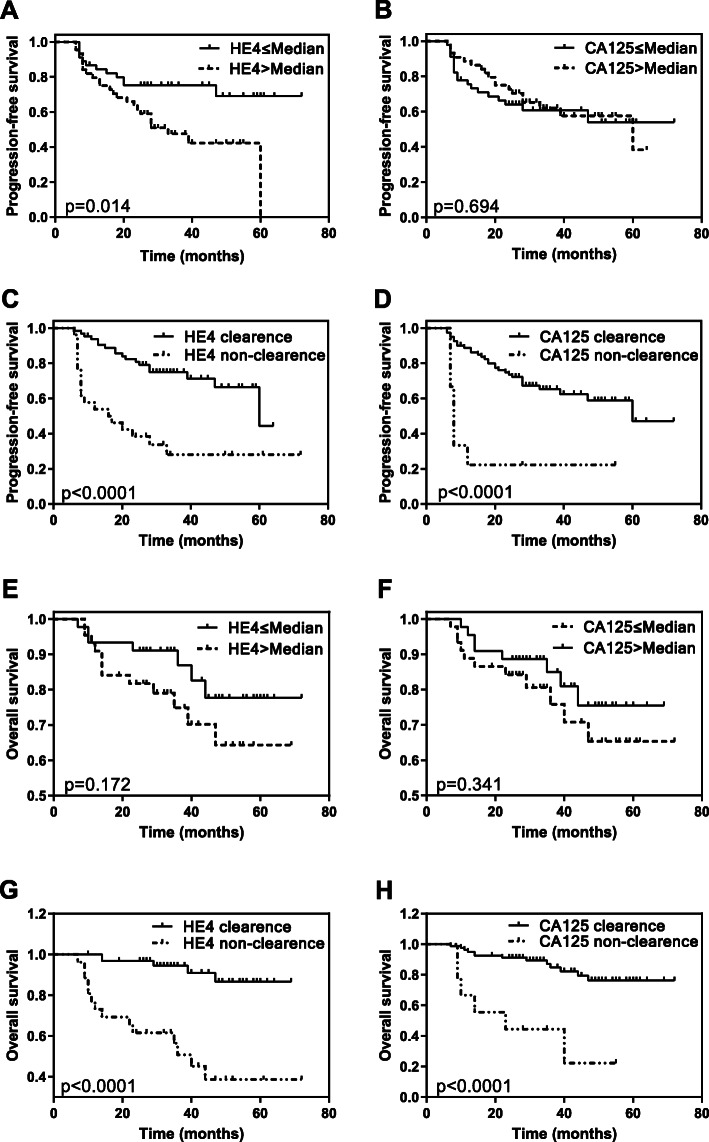
Correlation between the HE4 and CA125 level and OS/PFS. Kaplan-meier curve presenting the OS/PFS with different stratification of HE4 and CA125, including **a** The median of pretreatment HE4. **b** The median of pretreatment CA125. **c** The clearance of 3rd cycle chemotherapy. **d** The clearance of 1st cycle chemotherapy. **e** The median of pretreatment HE4. **f** The median of pretreatment CA125. **g** The clearance of 3rd cycle chemotherapy. **h** The clearance of 1st cycle chemotherapy

The Kaplan-Meier survival curve and a log rank test were conducted to analyze the relationship between patient’s PFS/OS and the value of HE4 and CA125 (Fig. 1). The result showed that the prolonged PFS and OS were significantly associated with the clearance of HE4 after 3rd cycle chemotherapy (*p*< 0.0001, *p*< 0.0001) and CA125 after 1st cycle chemotherapy (*p*< 0.0001, *p*< 0.0001). The pretreatment levels of HE4 had an impact on the PFS (*p*=0.014). However, there were no statistical significance between pretreatment levels of CA125 with PFS (*p*=0.694) and also no statistical significance between pretreatment HE4 and CA125 with OS (*p*=0.172, *p*=0.341).

Univariate and multivariate cox regress analysis were explored to analyze the widely recognized prognostic of EOC, as well as the early clearance of HE4 and CA125 (Table 4). The univariate analysis demonstrated a significant influence of stage, HE4-clearance after 3rd cycle of chemotherapy, and CA125-clearance after 1st cycle of chemotherapy with respect to both OS and PFS in patients. Multivariate analysis revealed that only the HE4-clearance after 3rd cycle of chemotherapy and CA125-clearance after 1st cycle of chemotherapy were significantly independently associated with OS. The prolongation of PFS was significantly influenced by the stage, HE4-median pretreatment, HE4-clearance after 3rd cycle of chemotherapy, and CA125-clearance after 1st cycle of chemotherapy.

**Table 4:** Univariate and multivariate analyses for OS and PFS

	Univariate analysis				Multivariate analysis			
	OS		PFS		OS		PFS	
	HR(95%CI)	*p*-value	HR(95%CI)	*p*-value	HR(95%CI)	*p*-value	HR(95%CI)	*p*-value
Age	1.033 (0.988–1.080)	0.155	1.001 (0.969–1.035)	0.931				
Grade 1 vs. grade 2,3	3.566 (0.474–26.854)	0.217	1.862 (0.570–6.080)	0.303				
Stage	1.946 (1.096–3.455)	0.023	2.092 (1.385–3.161)	0.000			1.877 (1.154–3.052)	0.011
Serous vs. other histopathology	1.114 (0.452–2.745)	0.814	0.815 (0.417–1.595)	0.550				
HE4-median pretreatment	1.893 (0.744–4.815)	0.181	2.325 (1.157–4.673)	0.018			2.315 (1.084–4.941)	0.030
HE4-clearance after 3rd cycle of chemotherapy	8.295 (2.984–23.057)	0.000	3.632 (1.877–7.027)	0.000	8.294 (2.980–23.085)	0.000	2.713 (1.361–5.407)	0.005
CA125-median pretreatment	0.645 (0.259–1.606)	0.346	0.878 (0.456–1.690)	0.697				
CA125-clearance after 1st cycle of chemotherapy	2.556 (1.4027–6.361)	0.044	2.435 (1.260–4.709)	0.008	2.549 (1.019–6.378)	0.045	3.853 (1.888–7.865)	0.000

## Discussion

To our knowledge, optimal tumor debulking and platinum response have been proven to be the most powerful prognostic factor for both overall survival and progression-free survival of ovarian cancer patients. To acquire optimal tumor debulking, multivisceral resection including en bloc resections with bowel resection, upper abdominal procedures, and extensive peritonectomy are required when necessary. Despite improvement in surgical techniques, there are still patients who are not obtain radical resection will develop platinum-resistance with poor prognosis. At present, CA125 are conventionally used in monitoring responds to surgery and chemotherapy for ovarian cancer patients. Data from GOG-182 showed that the median OS for patients whose CA125 value declined to normal level after 2nd cycle chemotherapy was 77.7 months, compared with 23.0 months for those who did not normalized, and improved PFS was observed in patients with CA125 value declined to normal level after 1st, 2nd or 3rd cycle chemotherapy compared to those who never normalized before 4th cycle chemotherapy [12].

HE4 is a novel tumor biomarker in EOC patients. The assessment of the prognostic significance of pretreatment HE4 has been described in many papers. However, few studies focused on the early clearance of HE4 after treatment in predicting prognosis of epithelial ovarian cancer. There were several results suggested that HE4 was mainly secreted by malignant ovarian cancer cell and tumor micro-environment [13, 18]. Therefore, the removal of the tumor and the response to treatment should correlate with the clearance of HE4.

A prospective study of Roberto Angioli suggested that HE4 reduction with above 47% at the third cycle of chemotherapy were more likely to be platinum sensitive. On the contrary, CA125 value did not correlate with platinum response [1]. In our recent study, we have demonstrated that single HE4 superior to CA125 in predicting platinum sensitivity. Our data showed that the maximum AUC value of HE4 alone was 0.779 (*p*=0.000) in predicting platinum sensitivity after the third cycle chemotherapy compared to the maximum AUC=0.731 of CA125 (*p*=0.004) after 1st cycle chemotherapy respectively. When the two biomarkers were combined, the result showed that when HE4 clearance after 3rd cycle chemotherapy or CA125 clearance after 1st cycle chemotherapy, the AUC, sensitivity and specificity were 0.788, 100 and 57.5% respectively. It means that 100% patients with platinum resistant could be identified through the both non-clearance of HE4 after 3rd cycle chemotherapy and CA125 after 1st cycle chemotherapy. When HE4 after 3rd cycle chemotherapy and CA125 after 1st cycle chemotherapy were both clearance, 94.5% individual with platinum sensitive could be recognized. Previous publications on the application of HE4 and CA125 as a method of predicting chemotherapy response have mainly focused on the pretreatment biomarker value [1, 4, 5, 20]. Anita et al. have reported that the predictive abilities with regard to platinum sensitivity for pretreatment HE4 was excellent with an AUC of 0.627, and poor for pretreatment CA125 with an AUC of 0.547 respectively [4]. Nassir et al. showed that high level of pretreatment HE4 and CA125 correlated significantly with a poor response to platinum based chemotherapy [20]. Few studies pay attention to the dynamic change of biomarker corresponding to treatment response during first-line treatment. Chen et al. concluded that the change of HE4 was more closely related to the chemotherapy response compared to the change of CA125 individually [3]. Vallius et al. presented their result that HE4 was a reliable serum tumor marker for monitoring treatment response in advanced EOC patients [27]. They focused on the postoperative level and the nadir value during postoperative chemotherapy with serum HE4 and CA125. Their result suggested that the single postoperative HE4 was associated to residual tumor after surgery, primary therapy outcome and PFS in both primary debulking surgery (PDS) and interval debulking surgery (IDS) patients. The single postoperative CA125 was associated to PFS after IDS but not for PDS. They also demonstrated that the combination of HE4 and CA125 nadir level predicted primary treatment outcome and PFS better than either alone. However, they didn’t refer the combination of the clearance speed of HE4 and CA125 in predicting treatment response and prognosis. In Pelissier’ report, the cut-off for CA125 of 35 UI/ml and combined with HE4 of 115 pmol/L after the 3rd cycle of neoadjuvant chemotherapy (NACT) has a sensitivity of 92.9% and a specificity of 68.7% (PPV=72.2% and NPV=91.7%) in predicting platinum sensitivity in a small cohort of 30 patients deemed inoperable with advanced EOC [21]. Our study is the first to evaluate the predicting effect of treatment response and platinum sensitivity with the combination of early clearance of HE4 and CA125 during first-line chemotherapy. Our results suggest that monitoring HE4 and CA125 during first-line chemotherapy should be recommended. It may help early identifying high-risk patients with platinum-resistant in EOC patients. In other words, for this part of high-risk patients with a slow decline in HE4 and CA125, the corresponding imaging evaluation or individualized treatment should be developed. At present, there are no clinical trials showing that modifying the treatment based on the unsatisfactory decline of serological tumor biomarkers after treatment can improve the outcome of patients. HE4 and CA125 appear to be able to select high-risk patients who demand further treatment base on their adverse features, but still need to be confirmed in larger studies.

Olivier Colomban et al. used a Kelim model [26], which characterize the CA125 elimination rate during the first 100 days of NACT and adjuvant chemotherapy, to assess the benefit in survival with bevacizumab addition for high-risk ovarian cancer patients in ICON-7 [9]. The result showed that only those high-risk patients with an unfavorable KELIM parameter less than 1.0 might have derived a benefit from bevacizumab when considering non-censored median survivals. With respect to HE4, previous study showed that patients with HE4 change of > 80% during NACT in advance high-grade serous ovarian cancer correlated with prolonged OS compared to change < 80% [28]. However, serum CA125 decline of > 80 and < 80% during NACT had no statistical significance in OS. Patients with CA125 logarithmic decrease or normalization within 1 month post-operative were correlated with better PFS and OS [25]. Our data also demonstrated that serum HE4 is a more promising biomarker in prognosis of EOC compared to CA125. In the ROC curve analysis, patients with HE4 level normalization or reduction above 90% after 3rd and 6th cycle chemotherapy significantly correlated with two-year PFS (AUC=0.707, *p*=0.001, and AUC=0.666, *p*=0.011). CA125 level normalization or reduction above 90% after 1st cycle chemotherapy correlated with two-year PFS, with a lower AUC of 0.648, *p*=0.023. When combination of the HE4 after 3rd cycle chemotherapy and CA125 after 1st cycle chemotherapy, it was shown that the AUC reached to 0.730 when one of them declined above 90% or normalized (*p*=0.000), reporting an 83.3% of sensitivity and a 62.7% of specificity. Approximately 83.3% of patient who relapsed or progressed in 2 years could be identified during first-line chemotherapy with a 62.7% of specificity based on the combination of HE4 and CA125 non-early clearance. Using biomarkers to identify high-risk patients was a simple and non-invasive way and may complement the current definition of high-risk patients.

The result of Kaplan-Meier survival curve and long rank test also demonstrated that both the clearance of HE4 after 3rd cycle chemotherapy and CA125 after 1st cycle chemotherapy were significantly correlation with the PFS and OS. Prolonged PFS was significantly impacted by the pretreatment HE4 value but not pretreatment CA125. As respected to OS, there were no significant correlation between pretreatment value of HE4 and CA125 in our results. Similar results were obtained in other research which analyzed the influence of the pretreatment HE4 on PFS [12, 14, 15, 24]. Amanda Fader et al. analyzed a group of 3686 patients with ovarian cancer and demonstrated that there was no difference in pretreatment CA125 with outcome [12]. However, patients with CA125 that normalized after 1st, 2nd, 3rd cycle of chemotherapy treatment were less likely to experience disease progression as compared to those of not normalization ones in Fader’ study. Anita et al. analyzed a group of 48 EOC patients treated with PDS and demonstrated that the prolongation of PFS and OS was significantly correlated with the pre-operative HE4 value [4]. Their result also concluded that the duration of OS was significantly influenced by the HE4 value after the third course of chemotherapy but not CA125, and the prolonged PFS was influenced by the CA125 value after the third course of chemotherapy treatment bot not the HE4. This result was different from ours. The reason for this problem may be due to its small sample size and selection bias.

This study was a retrospective character, single-center setting, relatively small number of patients, and lack of external validation, which may bias the results. A bigger prospective cohort or including more clinical covariate such as ECOG score, oncogene expression might be helpful to validate the result and provide more information. Therefore, further studies are needed to confirm these results.

## Conclusions

In conclusions, our findings revealed that the early clearance of HE4 and CA125 during first-line platinum chemotherapy were significantly associated with the platinum sensitivity and prognosis. Monitoring the dynamic value of both HE4 and CA125 during treatment might be helpful in future clinical practice.

## Data Availability

The data and materials which were analyzed and generated at the study are available from the corresponding author on reasonable request.

## Abbreviations

HE4: Human epididymis protein 4
CA125: Cancer antigen 125
EOC: Epithelial ovarian cancer
ADP: Adenosine Diphosphate
FIGO: The International Federation of Obstetrics and Gynecology Staging System
PFS: Progression-free survival
OS: Overall survival
CI: Confidence interval
GCIG: The Gynecology Cancer Intergroup
RECIST: The Response Evaluation Criteria in Solid Tumors
AUC: Area under the curve
ROC: Receive operator characteristics
PPV: Positive predictive value
NPV: Negative predictive value
PDS: Primary debulking surgery
IDS: Interval debulking surgery
NACT: Neoadjuvant chemotherapy
ECOG: Eastern Cooperative Oncology Group
